# Supplementary material for: Fe2O3/Porous Carbon Composite Derived from Oily Sludge Waste as an Advanced Anode Material for Supercapacitor Application
Source: Nanomaterials (Basel). 2022 Oct 28;12(21):3819. doi: 10.3390/nano12213819 (PMC9656837; doi:10.3390/nano12213819)
Supplement: Supplementary file 1 [file nanomaterials-12-03819-s001.zip › nanomaterials-1979237-supplementary.pdf]

Supplementary Materials

Fe_2O_3/Porous Carbon Composite Derived from Oily Sludge Waste as an Advanced Anode Material for Supercapacitor Application

Shubing Tian [1], Baoling Zhang [1], Dong Han [2], Zhiqiang Gong [3], Xiaoyu Li[1,2,*]

1. College of Mechanical and Electronic Engineering, Shandong University of Science and Technology, Qingdao 266590, China. College of New Energy, China University of Petroleum (East China), Qingdao 266580, China
2. State Grid Shandong Electric Power Research Institute, Jinan 250003, China

* Corresponding author's E-mail: lixy2018@sdust.edu.cn

Table S1 Elemental and proximate analyses of oily sludge

Items	Oil sludge				
**Elemental analysis** (*wt*%)	C	H	O	N	S
	16.38	4.25	8.91	0.32	2.34
**Proximate analysis** (*wt*%)	Moisture[a]	Volatile matter[b]		Fixed carbon[a]	Ash[a]
	16.61	27.38		4.82	51.19

[a] As received.

[b] Dry basis.

Table S2 Pore structure parameters of samples

**Sample**	S_{BET} **[m² g⁻¹]**	S_{mirco} **[m² g⁻¹]**	S_{meso} **[m² g⁻¹]**	V_{pore} **[cm³ g⁻¹]**	V_{mirco} **[cm³ g⁻¹]**	V_{meso} **[cm³ g⁻¹]**
FPC-90	851.3	363.7	487.6	0.739	0.269	0.470
FPC-180	635.2	337.7	297.5	0.382	0.236	0.146
α-Fe_2O_3	168.6	21.65	146.95	0.248	0.015	0.233

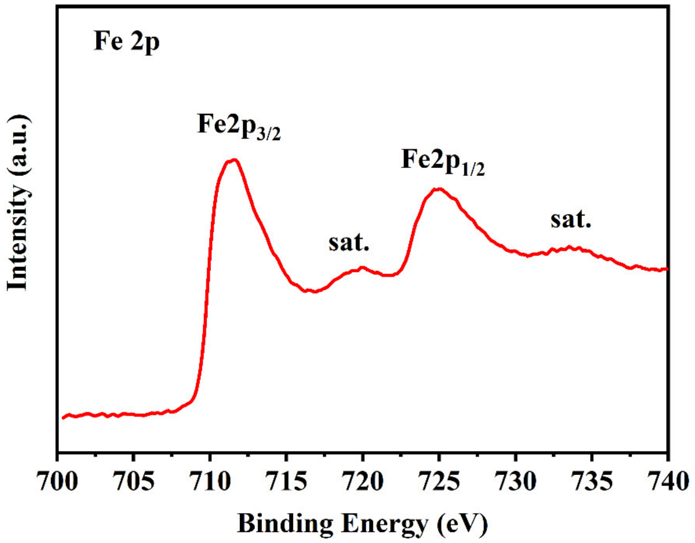

Figure S1 High-resolution of XPS spectrum of Fe2p

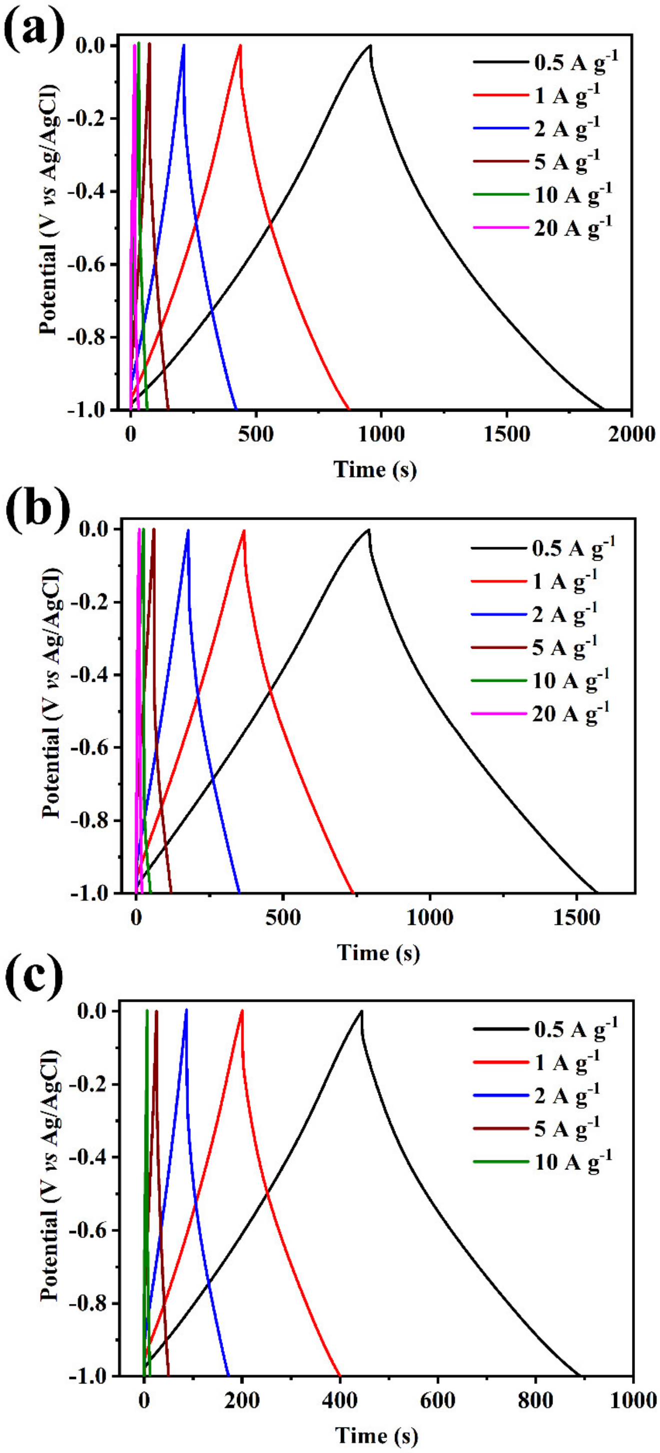

Figure S2 GCD curves of FPC composites (a) FPC-90, (b) FPC-180, (c) α-Fe_2O_3 at different current densities

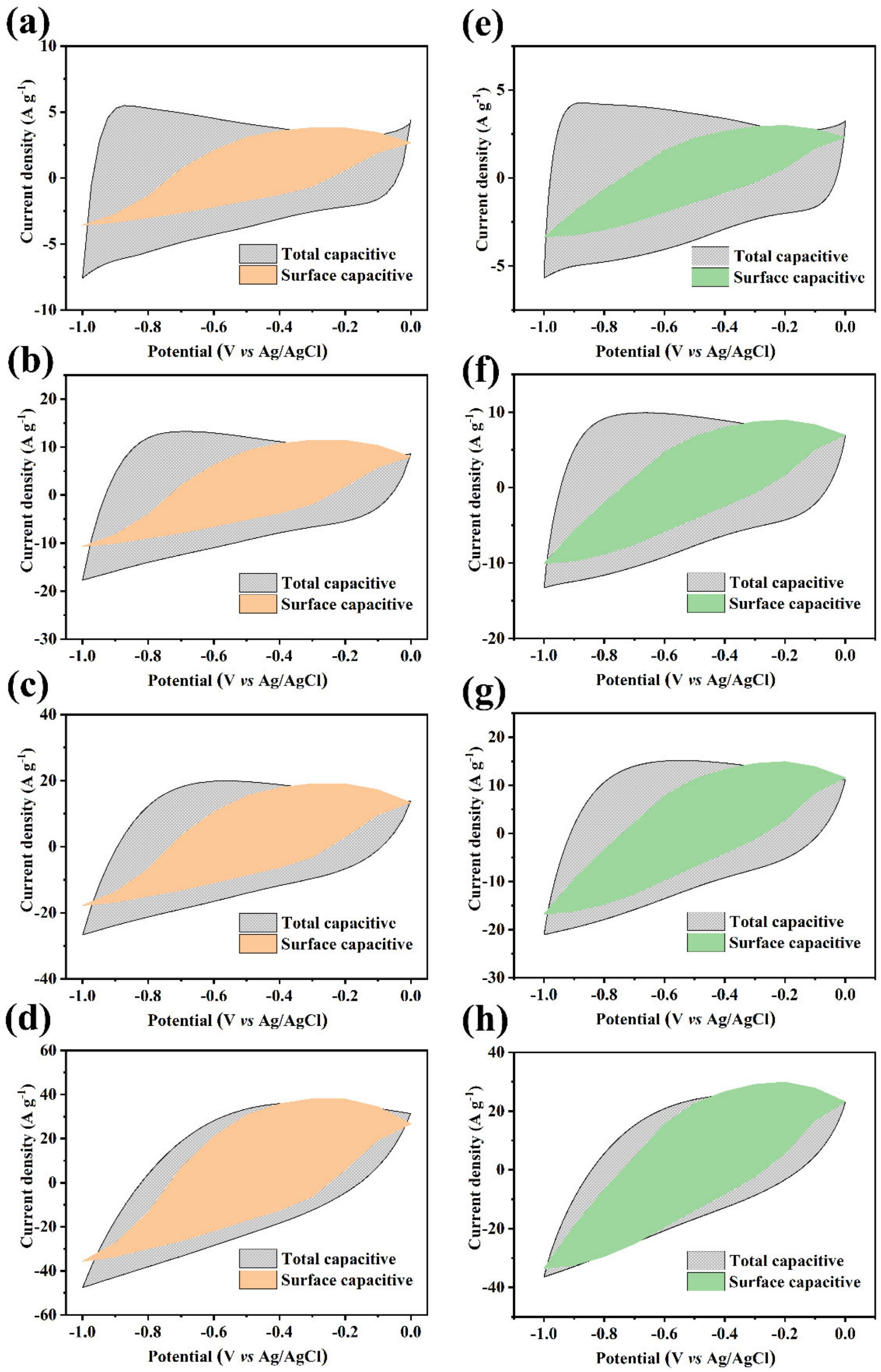

Figure S3 The charge storage contributions of FPC-90 (a, b, c, d) and FPC-180 (e, f, g, h) at different scan rates

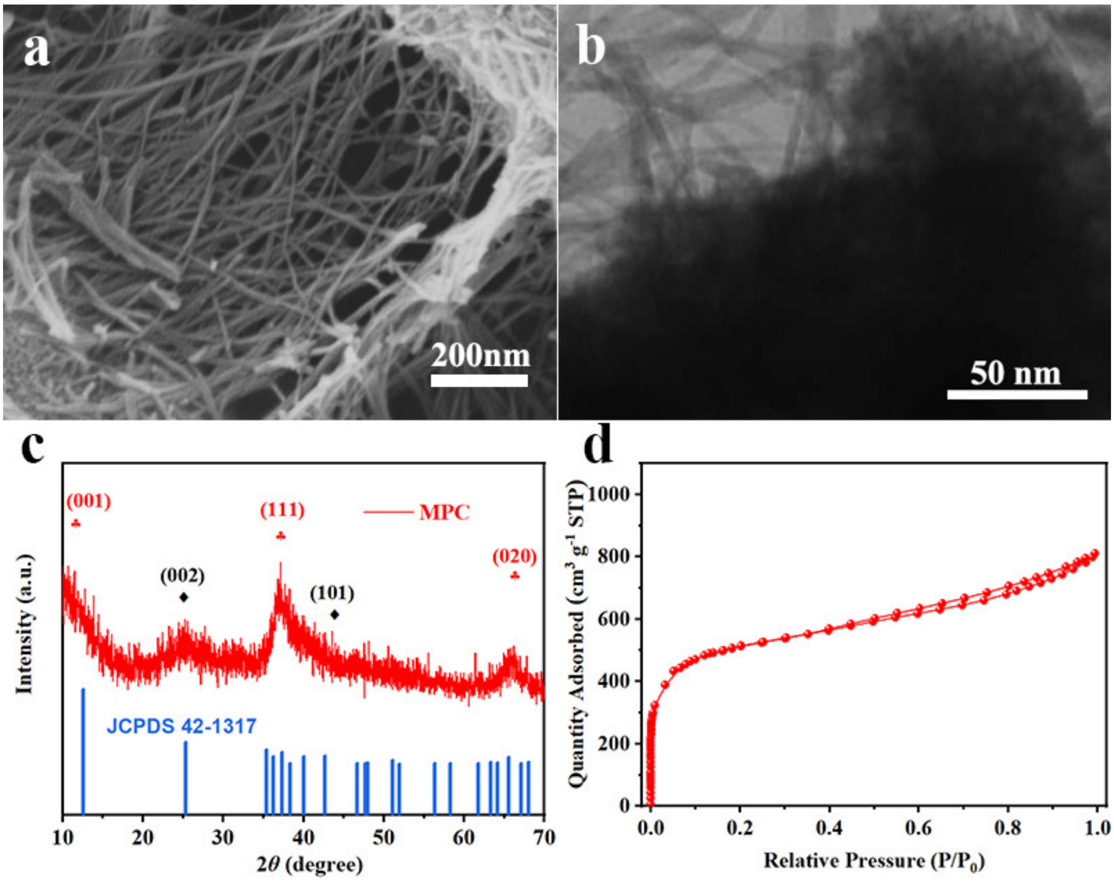

Figure S4 Characterization of MPC cathode material. (a) SEM image, TEM image, XRD pattern, and BET isotherm of MPC samples.

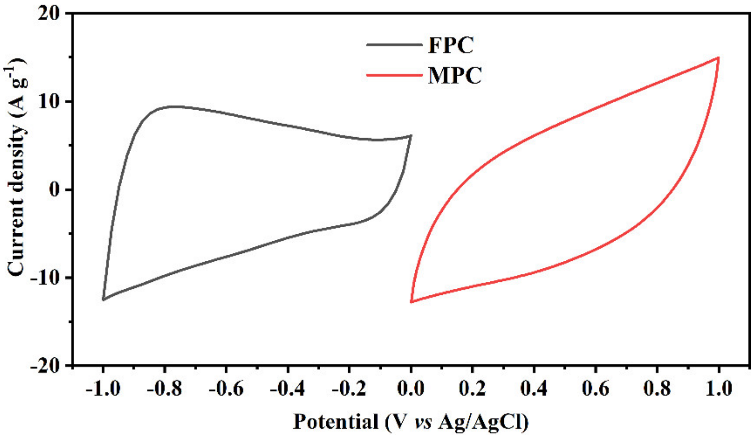

Figure S5 Comparative CV curves of MPC and FPC electrodes

For an ASC device, charge storage on the cathode and anode will be balance and follow the relationship of $Q_{cathode}=Q_{anode}$. The mass ratio of active material on both electrodes for the optimum performance satisfied the following equation:

$$\frac{m_{cathode}}{m_{anode}}=\frac{C_{anode}\times V_{anode}}{C_{cathode}\times V_{cathode}}$$

The overlaid CV curves of MPC and FPC measured on three electrode systems at a scan rate of 20 mV s^{-1} are illustrated in Fig. S5. As observed from the curves, the potential window of cathode (MPC) and anode (FPC) is in the range of 0-1.0 V and -1.0-0 V, respectively.

The specific capacitance of cathode and anode could be obtained by integrating these two CV curves. Therefore, the mass ratio of these two electrodes was calculated as about 1:1 according to the above charge balance equation.

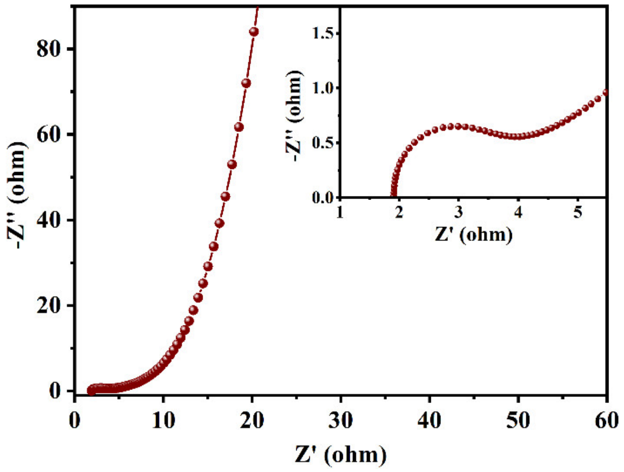

Figure S6 Nyquist plot of the ASC device

Table S3 Specific capacitance of recently published Fe_2O_3/Carbon composites

Active material	Electrolyte	Potential (V)	Capacitance (F g^{-1})
**Fe_2O_3/HPC in this work**	**1 M Na_2SO_4**	**-1.0 ~ 0 V *vs* Ag/AgCl**	**465 F g^{-1} at 0.5 A g^{-1}**
Fe_2O_3/ordered mesoporous carbon[1]	1 M Na_2SO_3	-1.0 ~ -0.2 V *vs* Ag/AgCl	235 F g^{-1} at 0.5 A g^{-1}
Fe_2O_3/CNT[2]	2 M KCl	-1.0 ~ 0 V *vs* Ag/AgCl	296.3 F g^{-1} at 5 mV s^{-1}
α-Fe_2O_3/Graphene[3]	1 M Na_2SO_4	-1.0 ~ -0.3 V *vs* Ag/AgCl	343.7 F g^{-1} at 3 A g^{-1}
α-Fe_2O_3/Graphene[4]	1 M Na_2SO_4	-1.2 ~ -0.2 V *vs* Ag/AgCl	306.9 F g^{-1} at 3 A g^{-1}
Fe_2O_3/Graphene[5]	2 M KOH	-1.0 ~ 0 V *vs* SCE	264 F g^{-1} at 2.5 A g^{-1}
Fe_2O_3/N-doped Graphene[6]	1 M Na_2SO_4	-1.1 ~ -0.1 V *vs* SCE	260.1 F g^{-1} at 2 A g^{-1}
α- Fe_2O_3/Graphene aerogel[7]	0.5 M Na_2SO_4	-0.8~0.8 V *vs* SCE	81.3 F g^{-1} at 1 A g^{-1}
Fe_2O_3 nanodots/N-doped Graphene[8]	2 M KOH	-1.0~0 V *vs* SCE	274 F g^{-1} at 1 A g^{-1}

Table S4 Performance of Fe_2O_3-based electrodes in asymmetric supercapacitors

Cathode	Anode	Voltage	Specific capacitance	Energy density
**MnO_2/HPC**	**Fe_2O_3/HPC**	**0-2.0 V**	**130.1 F g^{-1} at 0.5 A g^{-1}**<br>**81 F g^{-1} at 20 A g^{-1}**	**72.3 W h kg^{-1} at 500 W kg^{-1}**
MnO_2/m-rGO	Fe_2O_3/m-rGO[9]	0-1.8 V	73.9 F g^{-1} at 16.7 A g^{-1}	41.7 W h kg^{-1} at 13.5 kW kg^{-1}
GF/$CoMoO_4$	GF-CNT/Fe_2O_3[10]	0-1.6 V	115.5 F g^{-1} at 14 A g^{-1}	74.7 W h kg^{-1} at 1.4 kW kg^{-1}
MnO_2/FGS	Fe_2O_3/FGS[11]	0-2.0 V	73.2 F g^{-1} at 10 mV s^{-1}	50.7 W h kg^{-1} at 100 W kg^{-1}
3D HPC/$NiCo_2S_4$	3DHPC/Fe_2O_3[12]	0-1.6 V	101.2 F g^{-1} at 4 A g^{-1}	44.4 W h kg^{-1} at 1.62 kW kg^{-1}
NiO nanosheets	Fe_2O_3 NR[13]	0-1.8 V	57.2 F g^{-1} at 1 A g^{-1}	12.4 W h kg^{-1} at 312 W kg^{-1}
$NiCo_2O_4$/NiO	Fe_2O_3 NP[14]	0-1.6 V	57.2 F g^{-1} at 0.33 A g^{-1}	19 W h kg^{-1} at 157 W kg^{-1}
N-HPC	C@Fe_2O_3[15]	1-4 V	-	65 W h kg^{-1} at 368 W kg^{-1}
MnO_2/CNTs	Fe_2O_3/CNTs[16]	0-2.0 V	82.4 F g^{-1} at 0.1 A g^{-1}	45.8 W h kg^{-1} at 0.41 kW kg^{-1}
$ZnCo_2O_4$@MnO_2	Porous α-Fe_2O_3[17]	0-1.3 V	161 F g^{-1} at 2.5 mA cm^{-2}	35 W h kg^{-1} at 163 W kg^{-1}
$CuCo_2O_4$/CuO	Fe_2O_3/rGO[18]	0-1.6 V	83 F g^{-1} at 0.5 A g^{-1}	33 W h kg^{-1} at 200 W kg^{-1}
$CoMoO_4$/$NiMoO_4 \cdot xH_2O$	Fe_2O_3[19]	0-1.6 V	153.6 F g^{-1} at 1 A g^{-1}	41.8 W h kg^{-1} at 700 W kg^{-1}
$Ni_3(PO_4)_2$@GO	Fe_2O_3@GO[20]	0-1.6 V	175 F g^{-1} at 0.5 A g^{-1}	67.2 W h kg^{-1} at 200 W kg^{-1}

Reference

1. Lin, Y.; Wang, X. Y.; Qian, G.; Watkins, J. J., Additive-Driven Self-Assembly of Well-Ordered Mesoporous Carbon/Iron Oxide Nanoparticle Composites for Supercapacitors. *Chemistry of Materials* **2014,** 26, (6), 2128-2137.
2. Cheng, X. P.; Gui, X. C.; Lin, Z. Q.; Zheng, Y. J.; Liu, M.; Zhan, R. Z.; Zhu, Y.; Tang, Z. K., Three-dimensional alpha-Fe2O3/carbon nanotube sponges as flexible supercapacitor electrodes. *Journal of Materials Chemistry A* **2015,** 3, (42), 20927-20934.
3. Yang, S.; Song, X.; Zhang, P.; Gao, L., Heating-rate-induced porous alpha-Fe2O3 with controllable pore size and crystallinity grown on graphene for supercapacitors. *ACS applied materials & interfaces* **2015,** 7, (1), 75-9.
4. Yang, S.; Song, X.; Zhang, P.; Sun, J.; Gao, L., Self-Assembled alpha-Fe2O3 mesocrystals/graphene nanohybrid for enhanced electrochemical capacitors. *Small* **2014,** 10, (11), 2270-9.
5. Wu, J.; Zhou, A.; Huang, Z.; Li, L.; Bai, H., A Facile Method to Prepare Three-Dimensional Fe2O3/Graphene Composites as the Electrode Materials for Supercapacitors. *Chinese Journal of Chemistry* **2016,** 34, (1), 67-72.
6. Zhao, P.; Li, W.; Wang, G.; Yu, B.; Li, X.; Bai, J.; Ren, Z., Facile hydrothermal fabrication of nitrogen-doped graphene/Fe2O3

composites as high performance electrode materials for supercapacitor. *Journal of Alloys and Compounds* **2014,** 604, 87-93.

7. Song, Z.; Liu, W.; Xiao, P.; Zhao, Z.; Liu, G.; Qiu, J., Nano-iron oxide (Fe2O3)/three-dimensional graphene aerogel composite as supercapacitor electrode materials with extremely wide working potential window. *Materials Letters* **2015,** 145, 44-47.

8. Liu, L.; Lang, J. W.; Zhang, P.; Hu, B.; Yan, X. B., Facile Synthesis of Fe2O3 Nano-Dots@Nitrogen-Doped Graphene for Supercapacitor Electrode with Ultralong Cycle Life in KOH Electrolyte. *ACS applied materials & interfaces* **2016,** 8, (14), 9335-9344.

9. Yang, M.; Lee, K. G.; Lee, S. J.; Lee, S. B.; Han, Y. K.; Choi, B. G., Three-Dimensional Expanded Graphene-Metal Oxide Film via Solid-State Microwave Irradiation for Aqueous Asymmetric Supercapacitors. *ACS applied materials & interfaces* **2015,** 7, (40), 22364-22371.

10. Guan, C.; Liu, J. L.; Wang, Y. D.; Mao, L.; Fan, Z. X.; Shen, Z. X.; Zhang, H.; Wang, J., Iron Oxide-Decorated Carbon for Supercapacitor Anodes with Ultrahigh Energy Density and Outstanding Cycling Stability. *Acs Nano* **2015,** 9, (5), 5198-5207.

11. Xia, H.; Hong, C. Y.; Li, B.; Zhao, B.; Lin, Z. X.; Zheng, M. B.; Savilov, S. V.; Aldoshin, S. M., Facile Synthesis of Hematite Quantum-Dot/Functionalized Graphene-Sheet Composites as Advanced Anode

Materials for Asymmetric Supercapacitors. *Advanced Functional Materials* **2015,** 25, (4), 627-635.

12. Fan, H. L.; Liu, W.; Shen, W. Z., Honeycomb-like composite structure for advanced solid state asymmetric supercapacitors. *Chemical Engineering Journal* **2017,** 326, 518-527.

13. Zhang, S. W.; Yin, B. S.; Wang, Z. B.; Peter, F., Super long-life all solid-state asymmetric supercapacitor based on NiO nanosheets and alpha-Fe2O3 nanorods. *Chemical Engineering Journal* **2016,** 306, 193-203.

14. Shanmugavani, A.; Selvan, R. K., Microwave assisted reflux synthesis of NiCo2O4/NiO composite: Fabrication of high performance asymmetric supercapacitor with Fe2O3. *Electrochimica Acta* **2016,** 189, 283-294.

15. Yu, X. L.; Deng, J. J.; Zhan, C. Z.; Lv, R. T.; Huang, Z. H.; Kang, F. Y., A high-power lithium-ion hybrid electrochemical capacitor based on citrate-derived electrodes. *Electrochimica Acta* **2017,** 228, 76-81.

16. Gu, T. L.; Wei, B. Q., High-performance all-solid-state asymmetric stretchable supercapacitors based on wrinkled MnO2/CNT and Fe2O3/CNT macrofilms. *Journal of Materials Chemistry A* **2016,** 4, (31), 12289-12295.

17. Ma, W.; Nan, H.; Gu, Z.; Geng, B.; Zhang, X., Superior performance asymmetric supercapacitors based on ZnCo2O4@MnO2 core-shell electrode. *Journal of Materials Chemistry A* **2015,** 3, (10), 5442-5448.

18. Wang, Y. D.; Shen, C.; Niu, L. Y.; Li, R. Z.; Guo, H. T.; Shi, Y. X.; Li, C.; Liu, X. J.; Gong, Y. Y., Hydrothermal synthesis of CuCo2O4/CuO nanowire arrays and RGO/Fe2O3 composites for high-performance aqueous asymmetric supercapacitors. *Journal of Materials Chemistry A* **2016,** 4, (25), 9977-9985.

19. Wang, J.; Zhang, L. P.; Liu, X. S.; Zhang, X.; Tian, Y. L.; Liu, X. X.; Zhao, J. P.; Li, Y., Assembly of flexible CoMoO4@NiMoO4 center dot xH(2)O and Fe2O3 electrodes for solid-state asymmetric supercapacitors. *Scientific reports* **2017,** 7.

20. Li, J. J.; Liu, M. C.; Kong, L. B.; Wang, D.; Hu, Y. M.; Han, W.; Kang, L., Advanced asymmetric supercapacitors based on Ni-3(PO4)(2)@GO and Fe2O3@GO electrodes with high specific capacitance and high energy density. *Rsc Advances* **2015,** 5, (52), 41721-41728.
